# A temporary decrease in twitch response following reversal of rocuronium-induced neuromuscular block with a small dose of sugammadex in a pediatric patient

**DOI:** 10.1007/s00540-013-1688-3

**Published:** 2013-08-21

**Authors:** Hajime Iwasaki, Kenichi Takahoko, Shigeaki Otomo, Tomoki Sasakawa, Takayuki Kunisawa, Hiroshi Iwasaki

**Affiliations:** Department of Anesthesiology and Critical Care Medicine, Asahikawa Medical University, 2-1-1-1 Midorigaoka-higashi, Asahikawa, Hokkaido 078-8510 Japan

**Keywords:** Sugammadex, Rocuronium, Pediatric, Neuromuscular monitoring

## Abstract

We report a temporary decrease in twitch response following reversal of rocuronium-induced neuromuscular block with a small dose of sugammadex in our dose-finding study in pediatric patients. A 19-month-old female infant (9.6 kg, 80 cm) was scheduled for elective cheiloplasty surgery. Anesthesia was induced with nitrous oxide 50 % and sevoflurane 5 % and maintained with air, oxygen, sevoflurane 3 %, and fentanyl (total, 3 μg/kg). Neuromuscular monitoring was performed at the adductor pollicis muscle after induction of anesthesia but before the administration of rocuronium. Total dose of rocuronium during the surgery was 0.9 mg/kg. Neuromuscular block was reversed with 0.5 mg/kg sugammadex when one response was observed with post-tetanic count stimulation. Twitch responses after sugammadex administration showed a temporary decrease after its initial recovery. Maximum decreases in twitch responses were observed 17 min after initial dose of sugammadex. Twitch responses recovered to their control values after additional doses of 3.5 mg/kg sugammadex (4 mg/kg in total). Time from sugammadex administration to maximum decreases in twitch responses is earlier than has been reported in adults (20–70 min). It is demonstrated that following neuromuscular block reversal with insufficient dose of sugammadex, there is a possibility of the recurrence of residual paralysis within less than 20 min in pediatric patients.

## Introduction

Sugammadex, a modified γ-cyclodextrin, specifically antagonizes the neuromuscular block of steroidal neuromuscular blocking agents such as rocuronium and vecuronium [1]. Its efficacy in infants and children is reported to be similar to adults in a Phase III A study [2]. Moreover, sugammadex can reverse even a profound rocuronium-induced neuromuscular block [3]. However, the optimal dose of sugammadex to reverse profound neuromuscular block in pediatric patients has not been determined. There are risks of recurarization and recurrence of neuromuscular block when an insufficient dose of sugammadex was administered. It has been reported that recurrence of neuromuscular block was observed by using a small dose of sugammadex in adult patients [4, 5]. We describe a case of temporary decreases in twitch height and train-of-four (TOF) ratio after reversal of rocuronium-induced neuromuscular block with a small dose of sugammadex in our sugammadex dose-finding study in pediatric patients.

## Case reports

After informed consent, the patient’s parents agreed to participate in our dose-finding clinical study of sugammadex in pediatric patients approved by our medical ethics committee.

A 19-month-old female infant (9.6 kg, 80 cm, ASA Class 1) was scheduled for elective cheiloplasty surgery. She had no neuromuscular disorders and had not received any drug known to affect neuromuscular junction. The patient received no premedication.

Anesthesia was induced with nitrous oxide 50 % and sevoflurane 5 %, and maintained with air, oxygen, sevoflurane 3 %, and fentanyl (total 3 μg/kg). An intravenous cannula was inserted on the dorsum of the hand after the induction. Neuromuscular monitoring at the adductor pollicis muscle was performed by using TOF-Watch SX (Organon, Dublin, Ireland) acceleromyography after induction of anesthesia but before the administration of rocuronium. The transducer was attached to the thumb with tape on the opposite side of the intravenous cannula. After calibration and stabilization of the twitch responses, 0.6 mg/kg rocuronium was administered. The trachea was intubated after the disappearance of twitch responses of the TOF stimulation. An additional dose of 0.3 mg/kg rocuronium was administrated once to maintain neuromuscular block at less than two twitches of TOF. Peripheral skin temperature was kept above 34 °C by using a warming mattress. End-tidal sevoflurane and CO_2_ were kept constant until full recovery of neuromuscular block after surgery.

**Fig. 1:**
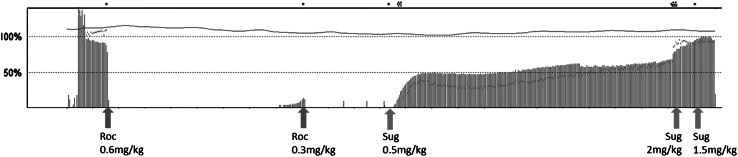
Progress of T_1_ height and train-of-four (TOF) ratio during surgery. A temporary decrease in twitch response after administration of a small dose of sugammadex (0.5 mg/kg) was observed. Initial dose of sugammadex was administered when one twitch response was observed with post-tetanic count. Additional doses of sugammadex (2 and 1.5 mg/kg) were administered before extubation. *Solid blue lines* represent the first twitch of the train-of-four (TOF); *red dots* represent TOF ratio. *Roc* rocuronium, *Sug* sugammadex

When the delicate part of the surgery was finished, the neuromuscular monitor showed one twitch response in post-tetanic count stimulation (1 PTC), and the neuromuscular block was reversed with 0.5 mg/kg sugammadex. Total dose of rocuronium given during the surgery was 0.9 mg/kg. The recovery of the twitch height of the first twitch (T_1_ height) and the TOF ratio to 50 and 39 %, respectively, were within 6 min after sugammadex administration. However, temporary decreases in T_1_ height and TOF ratio were observed (Fig. 1). Maximum decrease was observed 17 min after the first sugammadex administration, and the values of T_1_ height and TOF ratio were 47 % and 29 %, respectively. T_1_ height and TOF ratio were the same values at 6 and 26 min after the first sugammadex administration. At the end of the surgery (57 min after the first sugammadex administration), an additional 2 mg/kg sugammadex was administered to complete the reversal of the neuromuscular block. TOF ratio recovered to 90 % at 46 s after the second sugammadex administration. However, at the same moment, T_1_ height did not recover to its control value. T_1_ height and TOF ratio recovered to their control values after another dose of 1.5 mg/kg sugammadex (4 mg/kg in total). The trachea was extubated uneventfully after full recovery of the neuromuscular block.

## Discussion

The patient in this report was enrolled in our sugammadex dose-finding study for pediatric patients. The patient was in the group to which 0.5 mg/kg sugammadex was given to the patients at 1–2 PTC. Our protocol for tracheal extubation was to administer at least total 4 mg/kg of sugammadex (recommended dose to reverse neuromuscular block when 1–2 PTC) before that point. It is demonstrated that a temporary decrease in twitch response was observed during the time course in the reversal of rocuronium-induced neuromuscular block with a small dose of sugammadex in pediatric patients. The same event has been reported in adults [4, 5]. In these articles, the maximum decreases in T_1_ height and TOF ratio were observed approximately 20–70 min after sugammadex administration. In our patient, time from the small dose of sugammadex administration to maximum decreases in T_1_ height and TOF ratio was 17 min, respectively. We demonstrate that a temporary decrease in twitch response also occurs in pediatric patients after a small dose of sugammadex administration. These phenomena may be explained by redistribution of rocuronium from peripheral to central and effect-site compartments. Also, the time from sugammadex administration to maximum decrease of twitch response in pediatric patients was earlier than has been reported in adults. These time differences may be produced by the differences of pharmacokinetic and pharmacodynamic profiles of neuromuscular blocking agents between adults and pediatric patients [6]. It is clinically important to know that the time from sugammadex administration to maximum decrease in twitch response differs between pediatric patients and adults when the dose of sugammadex given is insufficient.

Not the T_1_ height but the TOF ratio recovered to its control value after the second dose of sugammadex (3.5 mg/kg in total). A total dose of 4 mg/kg of sugammadex was required for the recovery of T_1_ height and TOF ratio to their control values. It has been reported that full recovery of the TOF ratio is observed when T_1_ height is still depressed after the reversal of neuromuscular block by sugammadex [7, 8]. Therefore, it is important to confirm full and constant recovery of twitch height as well as recovery of TOF ratio ≥0.9. It is also demonstrated that at least 4 mg/kg sugammadex was required to reverse neuromuscular block when 1–2 PTC in this pediatric patient.

We conclude that a temporary decrease in twitch response may occur during reversal of rocuronium-induced neuromuscular block with a small dose of sugammadex in pediatric patients. Also, the time from small-dose sugammadex administration to maximum decrease of twitch response in pediatric patients was less than 20 min. Therefore, following neuromuscular block reversal with sugammadex, residual paralysis may occur earlier in pediatric patients than in adult patients.
